# Disability, ICF biopsychosocial model and burden of migraine

**DOI:** 10.1186/1129-2377-16-S1-A2

**Published:** 2015-09-28

**Authors:** Matilde Leonardi, Alberto Raggi, Licia Grazzi, Domenico D'Amico

**Affiliations:** SOSD Neurologia, Salute Pubblica, Disabilità, Fondazione IRCCS Istituto Neurologico Carlo Besta, Milan, Italy; UO Neuroalgologia e Centro Cefalee, Fondazione IRCCS Istituto Neurologico Carlo Besta, Milan, Italy

When defining the burden of migraine it is important to consider patients’ disability and clinical and public health perspectives. Migraine sufferers often have severe under recognized and underdiagnosed health burden and reductions in social activities and work capacity. Health professionals focus on diagnosis as a key element to effective treatments, however the majority of clinicians still tend to perceive migraine, and headache disorders in general, as minor complaints. Ten years ago a possible way to increase awareness and diminish the burden was described[1]. However epidemiological data of headache disorders, despite the international Lifting the Burden Campaign, is still scarce in many parts of the world and inconsistent because of the sampling frames and of how prevalence rates are defined and the physical, emotional, social and economic burdens of headaches are still poorly acknowledged.

Uncertainty about the prevalence distribution reflects that there is still need of instruments for classifying migraine in a comparable manner across populations and that more studies must be undertaken to classify the disability due to the disorder using reliable outcome measures[2]. Estimation of needs for health services, their costs and effectiveness require indicators that go beyond measures of death rates or of diagnosis alone, and include the “functioning” of people. The biopsychosocial model of the WHO Classification of Functioning, Disability and Health (ICF) provides the model, as well as the classification system, that allows to measure all dimensions of functioning and disability[3]. More than ten years of research with ICF in migraine sufferers shows that it allows data comparability and the evaluation of the role of environment. According to ICF construct any health condition, in an unfavourable environment, can cause disability. Environmental barriers for migraine sufferers are lack of health care facilities, of accurate diagnosis, of drugs, but also difficulty in being taken seriously. Steiner[4] drew attention to the high number of people with disability due to headache who do not receive health care. The barriers responsible for this might vary throughout the world, but poor awareness of headache in a context of limited resources generally was still constantly among them. Describing and accounting the burden of migraine worldwide is not enough anymore, we need to change our paradigm again and to move towards new pathways. The opportunity is provided by the biopsychosocial approach of the ICF. To reduce the burden of millions of migraine and headache sufferers once we cannot change the disease, we should change the environment and global efforts should focus on the new development of drugs but mainly on improving the response of health care systems.

## Conflict of interests

The authors certify that there is no actual or potential conflict of interest in relation to this article.
